# Tailoring the CFIR to Medication Adherence Interventions: A Delphi and Living Lab Study

**DOI:** 10.3390/pharmacy14030088

**Published:** 2026-06-22

**Authors:** Mirthe A. M. Oude Lansink, Bart J. F. van den Bemt, Caroline H. P. A. van de Steeg-van Gompel, Marcia Vervloet, Liset van Dijk, Charlotte L. Bekker

**Affiliations:** 1 Department of Research, Sint Maartenskliniek, Postbus 9011, 6500 GM Nijmegen, The Netherlands; 2 Department of Pharmacy, Sint Maartenskliniek, Postbus 9011, 6500 GM Nijmegen, The Netherlands; 3 Department of Pharmacy, Pharmacology and Toxicology, Radboudumc, Postbus 9101, 6500 HB Nijmegen, The Netherlands; charlotte.bekker@radboudumc.nl; 4 SIR Institute for Pharmacy Practice and Policy, Theda Mansholtstraat 5B, 2331 JE Leiden, The Netherlands; c.vandesteeg@sirstevenshof.nl; 5 Nivel Netherlands Institute for Health Services Research, Otterstraat 118, 3513 CR Utrecht, The Netherlands; m.vervloet@nivel.nl (M.V.); l.vandijk@nivel.nl (L.v.D.); 6 Department of Pharmacotherapy, -Epidemiology and -Economics, Groningen Research Institute of Pharmacy, Faculty of Science and Engineering, University of Groningen, A Deusinglaan 1, 9713 AV Groningen, The Netherlands

**Keywords:** medication adherence, determinant framework, community pharmacy, general practice, primary care, implementation

## Abstract

The implementation of medication adherence interventions is suboptimal. To guide implementation, this study aimed to tailor an existing implementation determinant framework to support the assessment of the implementability of such interventions in a specific context prior to implementation, and to investigate whether experts can assess in advance which determinants are important for implementing medication adherence interventions. In a Delphi study, experts rated determinants based on constructs of the Consolidated Framework for Implementation Research (CFIR) in terms of their importance for implementing medication adherence interventions. Determinants were then prospectively evaluated in four Dutch living labs implementing medication adherence interventions. The results were compared to assess agreement between expert opinion and real-world practice. Of 40 evaluated CFIR determinants, 16 were important in the majority of the living labs. These determinants concerned the inner setting, characteristics and roles of involved individuals, and implementation process domains of the CFIR. After comparing the prospective evaluation with Delphi results, expert opinions matched living lab observations for 18 out of 40 determinants (45%) regarding (un)importance. The CFIR was tailored to primary care medication adherence interventions based on practice observations, offering a potentially helpful framework to assess implementability of these interventions in specific contexts in advance. Determinant frameworks could benefit from incorporating real-world practice data.

## 1. Introduction

According to the existing literature, approximately 40% of patients on long-term treatment do not adhere to their medication regimen [1]. Effects of nonadherence are substantial as it culminates in worsened health conditions, more hospital admissions and increased healthcare spending [2]. Subsequently, the World Health Organization states that improving adherence to medical therapy for chronic diseases would result in substantial health and economic benefits [3]. Although effective interventions to improve medication adherence have been developed, these interventions are hardly implemented in routine clinical practice. The extent and speed of the implementation of medication adherence interventions seem to mirror other interventions in healthcare, where only 14% of effective interventions are implemented in daily practice, with an average duration of 17 years [4,5]. The slow adoption in clinical practice may be due to a predominant focus on efficacy rather than on implementation. Moreover, implementation challenges often arise as interventions and their implementation contexts are complex, requiring extensive change in individual roles, behaviors, relationships and processes [6]. Likely, not every intervention can be feasibly and sustainably embedded in a certain context.

To increase the implementation success of medication adherence interventions, insight into the factors hindering and facilitating implementation in clinical practice is essential. A framework specifying these factors, also referred to as implementation determinants, could inform the assessment of whether a medication adherence intervention is implementable in a certain healthcare context in advance. Additionally, such a framework could provide guidance in selecting suitable implementation strategies and inform necessary adaptations to either the intervention or the context in which the intervention is to be implemented [7]. Since implementation is generally a time-consuming process, an implementation framework tailored to medication adherence interventions based on observations in clinical practice may contribute to more efficient and successful implementation, ultimately benefiting clinicians, implementers, adopters, policymakers, and eventually patients.

Despite its potential value, the literature describing which determinants hinder and/or facilitate the implementation of medication adherence interventions is limited. The existing literature focuses on factors influencing medication adherence itself rather than on determinants that influence the implementation of interventions aimed at improving medication adherence [8]. Multiple frameworks aimed at guiding implementation do exist; however, these are not specific to medication adherence interventions [9,10]. An example is the Consolidated Framework for Implementation Research (CFIR), which describes and categorizes determinants that can influence implementation [11]. In addition, a study by Pringle and Coley proposes a framework for implementing medication adherence interventions in community pharmacies. However, this work is theory-based and addresses the implementation of healthcare interventions in general, rather than being informed by real-world data from clinical practice on the implementation of medication adherence interventions [12].

For multiple healthcare interventions, knowledge about implementation determinants is primarily derived from expert opinion rather than observations from clinical practice [13,14]. While expert opinions are valuable, it remains unclear to what extent they correspond to determinants that actually influence implementation in real-world clinical practice. Since implementation is context-dependent, arising from the interaction between an intervention and the context in which it is introduced, the importance of specific implementation determinants can be difficult to predict in advance [11]. This underscores the need to examine whether determinants considered important by experts align with those observed in clinical practice and to identify any differences between expert assessments and real-world observations.

To guide the implementation of medication adherence interventions in clinical practice, this study aims to tailor an existing implementation determinant framework, CFIR, to support the assessment of the implementability of primary care medication adherence interventions in a specific context in advance [9]. In addition, this study aimed to investigate whether experts can assess in advance which determinants are important for implementing medication adherence interventions, by comparing their assessment to real-world observations.

## 2. Materials and Methods

### 2.1. Study Design

This study consisted of two phases. First, a Delphi study was conducted between July 2020 and February 2021 to identify which determinants experts consider important for successful implementation of medication adherence interventions. The Delphi design was chosen as a consensus method to ensure that individual opinions remained independent and were not initially influenced by other individuals. Reporting of this first phase adheres to the Conducting and Reporting Delphi Studies (CREDES) [15].

In the second phase, implementation determinants were prospectively evaluated in four Dutch local living labs implementing various evidence-based effective medication adherence interventions between December 2020 and July 2022. Living labs are real-world test environments where healthcare providers, researchers, patients and other relevant stakeholders collaborate to create and refine healthcare innovations [16,17]. These living labs are part of “The Medication Adherence Knowledge and Expertise and Implementation Taskforce” (Make-It) program. This taskforce consists of medication adherence and implementation experts who aim to enhance the implementation of medication adherence interventions in clinical practice, by guiding, monitoring and evaluating their implementation within living labs [17]. A tailored framework for assessing the implementability of medication adherence interventions in a specific context was developed based on observed important implementation determinants. The Strengthening the Reporting of Observational Studies in Epidemiology (STROBE) Statement was applied for reporting this second phase [18]. To assess whether expert opinions align with real-world observations, the list of determinants deemed important by experts (phase 1) was compared to important implementation determinants observed in real-world practice (phase 2).

### 2.2. Theoretical Framework

To ensure that all implementation determinants were assessed, the CFIR 2022 version was used as a theoretical base for the study [9]. The CFIR is a comprehensive framework based on a comparison of a broad range of theories on effective implementation of interventions in practice, which consolidates determinants that are potentially relevant to a particular intervention and its context. The CFIR describes five major domains, each consisting of several determinants that can influence the effectiveness of implementation, including (1) the intervention, (2) the inner setting, comprising the features of the organization that implements the intervention, (3) the outer setting, comprising the features of the external context in which an organization resides, (4) the characteristics and roles of the individuals involved and (5) the implementation process.

### 2.3. Phase 1: Delphi Study

#### 2.3.1. Participants

A three-round Delphi study using online questionnaires was conducted with Dutch experts involved in medication adherence support or implementation, including general practitioners, community pharmacists, medical specialists, hospital pharmacists, nurses, patient representatives and researchers. The sample size was set at 30 to account for potential dropouts across rounds, ensuring that 20 experts participated in each Delphi round, in line with literature recommendations on the number of experts required to reach consensus [19,20]. Participants were identified through purposive sampling from the network of the Make-It consortium based on their profession and expertise, ensuring experts with diverse professional backgrounds and experiences were included [17]. Eligible participants were approached by email and asked to participate. Written informed consent was obtained prior to participation.

#### 2.3.2. Data Collection

**Figure 1:**
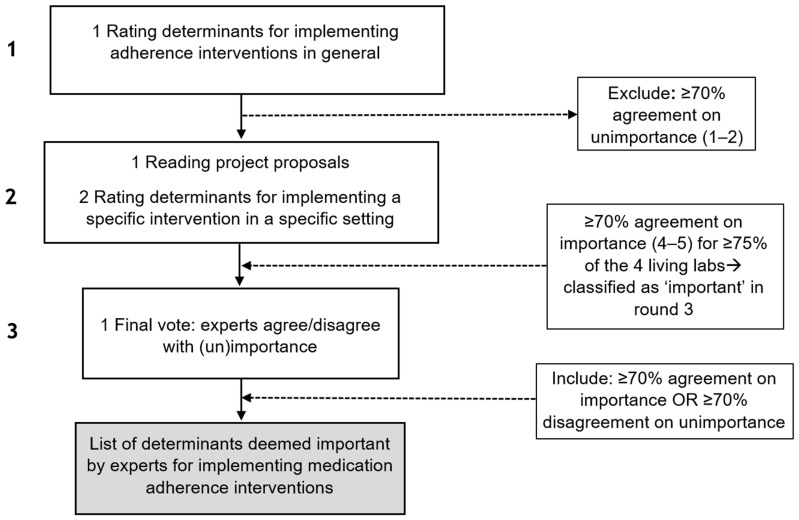
Flowchart describing three-round Delphi study.

In the first round of the Delphi study, participants rated all 40 determinants of the five domains of the CFIR on their perceived importance for implementing medication adherence interventions in general. Participants were provided with a short, translated determinant description. A five-point Likert scale (1 = very unimportant to 5 = very important) was used to rate importance. Determinants were excluded for the second round if there was ≥70% agreement on their unimportance (see Figure 1).

In the second round, participants rated the importance of the remaining determinants for anonymized project proposals describing a specific medication adherence intervention planned to be implemented in a particular living lab. This approach provided participants with a concrete example to facilitate a more accurate assessment. Each living lab had submitted these proposals as part of their grant application to ZonMw, the Netherlands Organization for Health Research and Development, which funded both the living labs and the Make-It consortium. The proposals outlined the context and characteristics of the living lab, along with the selected evidence-based medication adherence intervention. Determinants for which 70% or more experts agreed on their importance, for at least three of the four living labs, were classified as important determinants for the third round. If not, the determinants were classified as unimportant for the third round.

In the third round, participants gave their final vote on the importance of determinants, independent of specific living labs or interventions. Determinants classified as important after the second round were further evaluated with the yes/no question ‘Do you agree this determinant is important for the implementation of medication adherence interventions?’. Determinants classified as unimportant in the second round were assessed with the yes/no question ‘Do you agree this determinant is unimportant for the implementation of medication adherence interventions?’. If there was ≥70% disagreement on the unimportance of the determinant or ≥70% agreement on its importance in the third Delphi round, the determinant was included in the list of determinants that experts find important for implementing medication adherence interventions.

#### 2.3.3. Outcomes

The outcome of this first phase is a set of CFIR determinants considered important by experts for implementing medication adherence interventions. Determinants were included in this list if there was ≥70% disagreement in the third Delphi round on unimportance of the determinant or ≥70% agreement on importance.

#### 2.3.4. Data Analysis

Unimportance, as applied in the first Delphi round, was defined as a rating of 1 or 2 on the Likert scale. In the second Delphi round, importance was defined as a rating of 4 or 5 on the Likert scale. Descriptive statistics were used to check whether the level of (dis)agreement (70%) was obtained. Since the CFIR was updated in 2022, while the Delphi study had already been conducted using the 2009 version, the construct mapping document provided by Damschroder et al. was used to translate the determinants to the 2022 version [9]. For determinants that were not explicitly linked in the mapping document, a linkage was established if a match was identified based on determinant descriptions. This linkage was discussed until consensus was reached between MOL and CB, and was confirmed by other team members (BvdB, LvD). The linkage between the 2009 determinants and the CFIR determinants applied in this study is shown in Supplementary Table S1 [9,11].

### 2.4. Phase 2: Prospective Evaluation of Living Labs Implementing Medication Adherence Interventions

#### 2.4.1. Living Labs

The four Dutch living labs primarily consisted of community pharmacies and related general practices, in which pharmacy team members were the interventionists. The number of living labs was predetermined by the sponsor, ZonMw, from which they received funding for the implementation period [17]. Living labs were selected by a ZonMw committee based on quality and relevance of the project plan. Project plans included the intervention (chosen from a website presenting evidence-based medication adherence interventions; in some living labs, elements of different interventions were combined), contextual information and an implementation plan [21]. Table 1 describes the medication adherence intervention, setting and patient population for each living lab. The implementation process in the living labs was prospectively evaluated during a period of 1–2 years.

#### 2.4.2. Data Collection

Before and during implementation, data regarding the implementation process were collected via field notes, progress reports, and documentation of monthly meetings between the project team and Make-It members. In these monthly meetings, the implementation progress and experienced barriers and facilitators were discussed. Additionally, after 1–2 years of implementation, interviews were conducted by Make-It members with the project leaders to evaluate implementation outcomes, discuss barriers and facilitators encountered during the process, and to obtain additional insights from the project leaders’ perspectives. The interview guide was informed by the RE-AIM evaluation framework (Reach, Effectiveness, Adoption, Implementation and Maintenance) to ensure that all relevant implementation outcomes were addressed. A junior researcher prepared the initial draft of the guide, which was refined based on feedback from senior researchers CvdS, LvD, and MV. See Supplementary Table S2 for the interview guide [29].

#### 2.4.3. Outcomes

The outcome of this phase is the proportion of living labs in which each of the CFIR determinants played a role during the implementation. A determinant was considered to influence implementation if it was described at least once as acting as either a barrier or a facilitator for implementation.

#### 2.4.4. Data Analysis

Data analysis was performed with Atlas.ti version 9 after all data had been collected from the living labs. In the first step, documents were analyzed to identify implementation determinants. In the second step, the previously identified determinants were labeled with open codes and deductively analyzed using content analysis guided by CFIR (version 2022) [9]. The first step was conducted by a team of four senior researchers, one junior researcher and one master’s student in Pharmacy. Transcripts of project leader interviews were analyzed independently by pairs of researchers and then compared to discuss discrepancies. After reaching consensus on when a factor should be labeled as a determinant, determinants in the remaining documentations were identified by the junior researcher. In the second step, the determinants identified from one living lab were labeled with CFIR determinants by a junior researcher and reviewed by a senior researcher. Discrepancies were discussed until consensus was reached on how to place determinants within the CFIR. Subsequently, the junior researcher labeled and analyzed determinants from the remaining living labs, discussing any uncertainties with the senior researcher.

### 2.5. Developing the Tailored CFIR to Medication Adherence Interventions and Assessing Level of Agreement

#### 2.5.1. Outcomes

The main outcome of this study is the CFIR tailored to medication adherence interventions, which consists of all important implementation determinants observed during phase 2. To provide insights into how these determinants influenced the implementation in the living labs, the context of the determinants is described as well. A secondary outcome is the level of agreement between expert opinions (phase 1) and real-world observations (phase 2) on the importance of determinants for implementing medication adherence interventions. Agreement was presented as an overall percentage of determinants for which expert opinions matched real-world observation, and as a detailed figure showing the similarities and differences for each CFIR determinant at a living lab level.

#### 2.5.2. Data Analysis

Important implementation determinants were defined as determinants that were observed to either hinder or facilitate the implementation in at least three of the four living labs. The level of agreement between expert opinions and real-world observations in the living labs was assessed by dividing the number of determinants for which the expert opinions matched the observations (using the previously described definition for important determinants), by the total number of assessed CFIR determinants.

## 3. Results

### 3.1. Phase 1: Delphi Study

Out of the 29 approached Dutch medication adherence and/or implementation experts for the Delphi study, 18 consented to participate, with the following professional backgrounds: researcher (*n* = 4), (specialized) nurse (*n* = 4), primary care pharmacist (*n* = 3), outpatient pharmacist (*n* = 2), general practitioner (*n* = 2), medical specialist (*n* = 2), and hospital pharmacist (*n* = 1). Of these experts, 13 participated in the second round (5–8 experts per living lab), and 16 in the third round.

#### Important Implementation Determinants According to Expert Opinions

None of the 40 CFIR determinants were rated as unimportant by ≥70% of all the experts for implementing medication adherence interventions in the first round, resulting in all determinants being rated again in the second round (see Table 2). After reading the project proposals of living labs, experts rated the determinants on their importance for implementing the specific medication adherence intervention in the specific living lab. In this second round, 28 determinants were rated as important for at least three of the four living labs. In the third round, experts ultimately reached consensus that 28 determinants were important for implementing medication adherence interventions.

### 3.2. Phase 2: Prospective Evaluation of Living Labs Implementing Medication Adherence Interventions

Four living labs were prospectively evaluated to identify CFIR determinants influencing the implementation of medication adherence interventions in real-world practice. In addition to the documentation collected during the implementation process, evaluation interviews were conducted with project leaders with an average duration of 1 h. Results showed that 30 of the 40 assessed CFIR determinants influenced implementation in at least one of the four living labs. Among these, 10 determinants influenced implementation in four living labs, 6 determinants influenced implementation in three living labs, 5 in two living labs and 9 in one living lab.

### 3.3. CFIR Tailored to Medication Adherence Interventions

In phase 2, 16 determinants were identified as having influenced implementation in at least three of the four living labs. These determinants belong to the innovation, inner setting, roles of individuals, characteristics of individuals and the implementation process domains of the CFIR. Based on their influence observed in real-world practice, these determinants were included in the tailored framework (see Table 3). Determinants that were not included in the tailored framework are detailed in Supplementary Table S3.

The descriptions below provide insight into how determinants included in the tailored framework influenced implementation in the living labs, organized per CFIR domain.

#### 3.3.1. Innovation

The possibility to adapt the intervention was the only determinant of the innovation domain that influenced the implementation. This increased the willingness of the team to implement the intervention and increased the compatibility of the intervention with the existing working routine.

#### 3.3.2. Inner Setting

Multiple determinants of the inner setting were observed to influence the implementation. First, compatibility was a barrier when the intervention required additional actions outside the daily pharmacy routine. However, integrating the intervention into the pharmacy information system facilitated the implementation. Second, other activities, such as medication reviews, sometimes hindered implementation because they were prioritized due to reimbursement. Another barrier was a lack of available resources. Specifically, a lack of time had a substantial influence in pharmacies and general practices, frequently caused by staff shortages and a high workload.

Differing from expert opinions during the Delphi study, structural characteristics, communications, and culture within pharmacies and general practices was important as well. Structural characteristics include the organization of tasks and responsibilities. Specifically, the reallocation of tasks to pharmacy technicians or managers enabled pharmacists to dedicate more time to pharmaceutical care and project management. Additionally, the location of the pharmacy within a healthcare center alongside a general practice facilitated short communication lines. Lastly, regarding the culture within the living lab, pharmacy technicians mentioned feeling a hierarchical barrier when approaching general practitioners.

#### 3.3.3. Individuals—Roles

In contrast to expert opinions, implementation facilitators (i.e., Make-It guidance) and formally appointed implementation leads influenced the implementation in the living labs. Formally appointed leads and high-level leaders (i.e., project leads and pharmacists) were key facilitators, as they had the ability to influence the team’s motivation. Specifically, pharmacists who actively participated in delivering the intervention boosted overall motivation.

#### 3.3.4. Individuals—Characteristics

Capable and motivated team members were observed as key facilitators during implementation, and were also identified as such by experts. Healthcare providers with intrinsic motivation to care for patients were especially crucial, as they continued to deliver the intervention despite a high workload, whereas others neglected the intervention under these circumstances.

#### 3.3.5. Implementation Process

Aligning with expert opinions, all assessed implementation process determinants were observed to be important during implementation. Different methods were employed to engage healthcare providers to perform the intervention. These activities included a regular newsletter with updates, work meetings that solicited input from the entire team, and sharing success stories. Furthermore, all living labs continuously reflected and evaluated the implementation progress. Benchmarking, through comparison of results across pharmacies and/or general practices within a living lab, provided input for meetings in which successes, barriers, and tips were exchanged.

### 3.4. Level of Agreement Between Expert Opinions and Living Lab Observations

**Figure 2:**
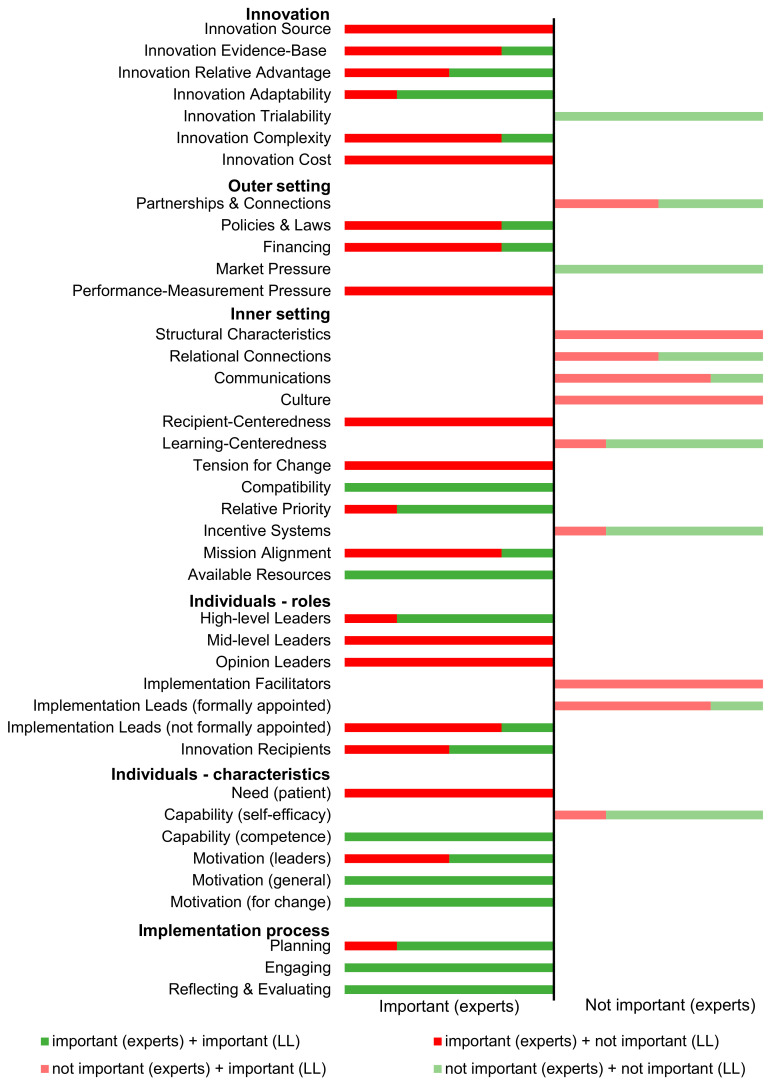
Comparison between expert opinions (phase 1) and living lab observations (phase 2). Bar lengths are indicative of the proportion of living labs for which the determinant was or was not observed. LL = living lab.

In the first phase of this study, 28 of the 40 determinants were ultimately considered important by the experts. Of these determinants, 11 were indeed observed to be important in at least three of the four living labs in the second phase. Twelve determinants were not considered important by experts in the first phase. Seven out of twelve were not observed to be important during the prospective evaluation. Ultimately, expert opinions matched real-world observations for 45% of the 40 evaluated determinants. The similarities and differences between expert opinions in phase 1 and real-world observations in phase 2 are presented in Figure 2.

## 4. Discussion

Our results suggest that the characteristics and roles of individuals, the organization itself and actions undertaken to stimulate the implementation process are at least important for implementing medication adherence interventions in the Dutch primary care setting. These determinants were therefore included in the CFIR tailored to medication adherence interventions. In addition, our results indicate that it is difficult for medication adherence and implementation experts to assess which determinants will influence the implementation of medication adherence interventions in advance. It is therefore essential to include information from real-world clinical practice in implementation frameworks and tools assessing implementability.

Upon comparing expert opinions to observations in the living labs regarding the importance of CFIR determinants for implementing medication adherence interventions, expert opinions aligned with observations regarding the importance of motivated and capable individuals. This is in line with the study by Shoemaker et al., in which characteristics of individuals was the most important CFIR domain for implementing medication management interventions [30]. Weir et al. also found the team members’ views and opinions on delivering the intervention to be important for implementing interventions in community pharmacies [31]. Additionally, expert opinions regarding the inner setting aligned with living lab observations on compatibility, relative priority and available resources. Compatibility of the intervention with pharmacy systems, lack of time and staff shortages have also been found to influence implementation in community pharmacies in the study by Weir et al. [31].

However, discrepancies were identified between experts’ opinions and living lab observations regarding the inner setting. Experts seemed to underestimate the importance of relatively softer characteristics of an organization, such as communications, culture and structural characteristics. This underestimation might be explained by the fact that the project proposals, on which the experts based their opinion regarding the importance of determinants for a specific context in the second Delphi round, did not contain such information. Thus, to facilitate the assessment of implementability in advance, project plans should include more information about the communication structure, organization of tasks and responsibilities, and if possible perceived working culture. This need for detailed context information in project plans has also been emphasized by Proctor et al. [32]. Furthermore, the importance of the intervention itself, as rated by experts, was not reflected in living lab observations. This discrepancy may be attributed to the living labs having the freedom to select which medication adherence intervention to implement, with costs already covered by project funding. Additionally, the living labs made adaptations to the interventions to ensure a better fit with their context. Indeed, innovation adaptability was noted to have influenced implementation in the living labs. Moreover, the outer setting, specifically determinants regarding policies and financing, may have had less influence due to the funding that the living labs already received. In situations where an intervention is not freely chosen and no funding is available, the innovation and outer setting domains might have a greater influence on the implementation of medication adherence interventions than observed in the living labs. More generally, the discrepancies between expert opinions and real-world observations could also be the result of the dynamic interaction between an intervention and its context, as determinants arise from the interaction between the two. This interaction can be difficult to predict in advance [11].

The observed discrepancies demonstrate that assessing implementability, thereby defining which determinants are important for implementing medication adherence interventions, can be challenging for experts. Real-world data should therefore play a more prominent role in the tailoring of determinant frameworks, as these are currently often based on expert opinions [13,14]. The experts’ challenges also highlight the added value of a tailored framework based on real-world data, as it provides guidance on which implementation determinants should be considered when evaluating implementability. To further facilitate this assessment, organizations should incorporate the determinants included in the tailored framework in their project plans and explicitly describe how they may act as barriers or facilitators within their setting. Together, this framework and more informative project plans can provide a more comprehensive assessment of the implementability of medication adherence interventions in specific contexts and thereby improve the chance at successful implementation. In addition to assessing implementability, our findings may also increase implementation success by tailoring strategies to overcome potential barriers, using the CFIR-ERIC matching tool [7]. For further research, it would be interesting to assess the applicability of our results in settings outside the Netherlands, as some determinants may be context-specific, especially those related to the outer setting [11].

A strength of this study is that it focuses on implementation determinants for the implementation of medication adherence interventions based on data obtained from real-world clinical practice. Furthermore, a key strength of the prospective evaluation is the fact that diverse medication adherence interventions were being implemented in four different living labs. Identifying a determinant that influenced implementation in the majority of these varied settings suggests that the determinant is important and may be crucial for the implementation of medication adherence interventions in different contexts. Another strength is the structured approach employed in terms of a Delphi design to obtain experts’ opinions on the importance of CFIR determinants for the implementation of medication adherence interventions. An additional strength is the inclusion of experts in medication adherence support or implementation with diverse backgrounds, thereby making the Delphi ratings more credible. However, several limitations should be acknowledged as well. For the second Delphi round, aimed at rating determinants for implementing a certain medication adherence intervention in a certain context, experts were provided with written information only. The project proposals they evaluated included mainly information on living lab characteristics and the chosen adherence intervention, and were less focused on implementation context. As described earlier, this could possibly explain why relatively softer characteristics of the inner setting domain were underestimated. Additionally, the living labs were early adopters, meaning the living labs and their project leaders had previous experience with implementing interventions in daily practice. Observed determinants could differ for organizations with less experience in implementing interventions. Another reason why our results may be less generalizable is that the living labs were located in primary care settings in the Netherlands. In other contexts, different determinants, particularly of the outer setting, may influence implementation. Furthermore, the design of the living labs, with funding and Make-It guidance, has likely influenced which determinants influenced implementation in the living labs, particularly those related to the outer setting. The fact that these determinants are not included in the tailored framework does not necessarily indicate that they would not impact implementation in other contexts. Consequently, while the tailored framework may provide useful guidance regarding intervention characteristics, inner setting, individual characteristics and roles, and implementation processes, implementers outside the Dutch context or without funding and implementation guidance should additionally evaluate which outer setting determinants may influence implementation in their local context. Lastly, the absence of determinants in the data from living labs obtained during prospective evaluation does not imply that these determinants were not relevant. Some facilitators may have been unconsciously included in the project and therefore not recognized as a determinant. Additionally, certain determinants may have had a strong influence but were not included in the tailored framework as they did not meet the criterion of having influenced implementation in at least three of the four living labs.

In conclusion, this study tailored the Consolidated Framework for Implementation Research to the implementation of medication adherence interventions in primary care based on observations from clinical practice. This tailored framework can be used to guide the assessment of medication adherence interventions’ implementability in specific settings in advance and subsequently improve implementation. Differences between real-world practice data and expert opinions suggest that implementation determinant frameworks could benefit from incorporating real-world practice data.

## Figures and Tables

**Table 1:** Description of living labs.

	Living Lab			
	A	B	C	D
**Intervention**	Animated patient information followed by telephone counseling intervention [22,23]	Annual consultation for patients who use chronic medication including preparation of this consultation by patients, based on three different interventions [24,25,26]	Telephone counseling of patients who may be non-adherent according to dispensing records [22,26]	Teach-back method and comprehensible drug label instructions [27,28]
**Setting and involved healthcare providers**	Outpatient pharmacy, 14 community pharmacies and collaborating general practitioners	Three community pharmacies and collaborating general practitioners	Five community pharmacies and cooperating general practitioners, a hospital and a home care organization	Two community pharmacies and cooperating general practitioners
**Patient population**	Patients with cardiovascular disease initiating treatment, after hospital discharge	Patients who use the automated refill service	Patients taking cardiovascular medication	All patients starting medication, of which 2/3 are estimated to have limited health literacy

**Table 2:** Responses of experts on (un)importance of CFIR determinants for implementing medication adherence interventions [9].

Determinants Based on CFIR Constructs (per Domain)	Delphi Round 1 Unimportant for Implementation?	Delphi Round 2 Important for Imple Mentation of Specific Intervention in Specific Context?	Delphi Round 3 Agreement on (Un)importance Yes/No
	Proportion of Experts Rating ‘Unimportant’ (*n* = 18)	Number of Living Labs for Which Experts Obtained ≥70% Agreement on Importance (*n* = 4)	Proportion of Experts That Agree (*n* = 16)
**Innovation**			
Innovation Source *	6%	4	81% (important)
Innovation Evidence-Base *	11%	3	100% (important)
Innovation Relative Advantage *	6%	4	94% (important)
Innovation Adaptability *	0%	4	88% (important)
Innovation Trialability	0%	1	63% (unimportant)
Innovation Complexity *	11%	3	100% (important)
Innovation Cost *	0%	3	94% (important)
**Outer setting**			
Partnerships & Connections	11%	1	100% (unimportant)
Policies & Laws *	0%	3	94% (important)
Financing *	0%	3	94% (important)
External Pressure—Market Pressure	17%	0	100% (unimportant)
External Pressure—Performance-Measurement Pressure *	0%	3	94% (important)
**Inner setting**			
Structural Characteristics ^a^	6%	2	94% (unimportant)
Relational Connections	6%	2	100% (unimportant)
Communications	6%	2	100% (unimportant)
Culture	11%	2	94% (unimportant)
Culture—Recipient-Centeredness *	0%	4	100% (important)
Culture—Learning-Centeredness	0%	2	69% (unimportant)
Tension for Change *	0%	3	94% (important)
Compatibility *	0%	3	81% (important)
Relative Priority *	0%	3	94% (important)
Incentive Systems	28%	0	81% (unimportant)
Mission Alignment *	0%	3	100% (important)
Available Resources ^a^*	0%	3	100% (important)
**Individuals—roles**			
High-level Leaders *	0%	4	100% (important)
Mid-level Leaders *	0%	4	100% (important)
Opinion Leaders *	0%	3	100% (important)
Implementation Facilitators	11%	0	88% (unimportant)
Implementation Leads (formally appointed)	0%	2	94% (unimportant)
Implementation Leads (not formally appointed) *	0%	4	100% (important)
Innovation Recipients *	0%	4	100% (important)
**Individuals—characteristics**			
Need (patient) *	0%	4	100% (important)
Capability (self-efficacy)	6%	2	81% (unimportant)
Capability (competence) *	11%	4	94% (important)
Motivation (leaders) *	0%	4	100% (important)
Motivation (general) *	11%	4	94% (important)
Motivation (for change) *	0%	4	94% (important)
**Implementation Process**			
Planning *	17%	3	81% (important)
Engaging ^a^*	0%	4	100% (important)
Reflecting & Evaluating ^a^*	0%	4	100% (important)

^a^ Subconstructs of these constructs were not separately rated in the Delphi, as the 2009 CFIR version did not divide the construct in subconstructs. * Determinant was ultimately considered important for the implementation of medication adherence intervention by the experts.

**Table 3:** CFIR tailored to implementation of medication adherence interventions.

CFIR Determinants (per Domain) Observed to Influence Implementation	Important Following Expert Opinions (Phase 1)
**Innovation**	
Innovation adaptability	Yes
**Inner setting**	
Structural Characteristics	No
Communications	No
Culture	No
Compatibility	Yes
Relative Priority	Yes
Available Resources	Yes
**Individuals—Roles**	
High-level leaders	Yes
Implementation Facilitators	No
Implementation Leads (formally appointed)	No
**Individuals—Characteristics**	
Capability (competence)	Yes
Motivation (general)	Yes
Motivation (for change)	Yes
**Implementation Process**	
Planning	Yes
Engaging	Yes
Reflecting & Evaluating	Yes

This tailored framework includes determinants that influenced implementation in at least three out of four living labs based on prospective evaluation (phase 2). The additional column indicates whether these observations matched with expert opinions (phase 1) [9].

## Data Availability

The original contributions presented in this study are included in the article and Supplementary Material. Further inquiries can be directed to the corresponding author.

## Supplementary Materials

The following supporting information can be downloaded at: https://www.mdpi.com/article/10.3390/pharmacy14030088/s1, Table S1: description of how the CFIR 2009 determinants (applied for phase 1—Delphi study) were linked to the CFIR determinants applied for phase 2 of our study (prospective evaluation), based on the CFIR 2022 version; Table S2: interview guide used for interviews with living lab project leaders; Table S3: evaluated CFIR constructs not included in the tailored framework.
