# Supplementary material for: Tailoring the CFIR to Medication Adherence Interventions: A Delphi and Living Lab Study
Source: Pharmacy (Basel). 2026 Jun 22;14(3):88. doi: 10.3390/pharmacy14030088 (PMC13306722; doi:10.3390/pharmacy14030088)
Supplement: Supplementary file 1 [file pharmacy-14-00088-s001.zip › pharmacy-4318796-supplementary.pdf]

Table S1. A description of how the CFIR 2009 determinants (applied for phase 1—Delphi study) were linked to the CFIR determinants applied for phase 2 of our study (prospective evaluation) based on the CFIR 2022 version.

		**Determinant linked according to**	
**CFIR 2009[1]**	**CFIR applied in this study, based on 2022 version[2]**	**Construct Mapping[2]**	**Discussion and consensus between MOL and CB**
Intervention Source	Innovation Source	x	
Evidence Strength & Quality	Innovation Evidence Base	x	
Relative Advantage	Innovation Relative Advantage	x	
Adaptability	Innovation Adaptability	x	
Trialability	Innovation Trialability	x	
Complexity	Innovation Complexity	x	
Design Quality and Packaging	Innovation Design[a]	x	
Cost	Innovation Cost	x	
Patient Need & Resources	Innovation Recipients	x	
	Need (Patients)	x	
	Recipient Centeredness	x	
Cosmopolitanism	Partnerships & Conditions	x	
External Policies & Incentives	Policies & Laws	x	
	Financing		x
	Performance-Measurement Pressure	x	
Peer Pressure	Market Pressure	x	
Structural Characteristics	Structural Characteristics	x	
Networks & Communications	Relational Connections	x	
	Communications	x	
Culture	Culture	x	
Learning Climate	Learning Centeredness	x	
Tension for Change	Tension for Change	x	
Compatibility	Compatibility	x	
Relative Priority	Relative Priority	x	
Organizational Incentives & Rewards	Incentive Systems	x	
Goals & Feedback	Mission Alignment	x	
Available Resources	Available Resources	x	

Access to Knowledge and Information	Access to Knowledge and Information[a]	x	
Leadership Engagement	High-level Leaders	x	
	Mid-level Leaders	x	
	Motivation (leaders)		x
Opinion Leaders	Opinion Leaders	x	
External Change Agents	Implementation Facilitators	x	
Formally Appointed Internal	Implementation Leads (formally appointed)	x	
Implementation Leaders & Champions			
Champions	Implementation Leads (not formally appointed)	x	
Self-efficacy	Capability (self-efficacy)		x
Other Personal Attributes	Capability (competence)		x
	Motivation (general)		x
Individual State of Change	Motivation (for change)		x
Planning	Planning	x	
Engaging	Engaging	x	
Executing	Doing[a]	x	
Reflecting & Evaluating	Reflecting & Evaluating	x	

[a] Determinants were not analyzed correctly after the second Delphi round, which resulted in incorrect classifications (e.g., important instead of unimportant) in the third Delphi round. As a result, these determinants were excluded from the tailored framework.

References

1. Damschroder LJ, Aron DC, Keith RE, Kirsh SR, Alexander JA, Lowery JC. Fostering implementation of health services research findings into practice: a consolidated framework for advancing implementation science. *Implement Sci.* **2009**;*4*:50.
2. Damschroder LJ, Reardon CM, Widerquist MAO, Lowery J. The updated Consolidated Framework for Implementation Research based on user feedback. *Implement Sci*. **2022** *17(1):*75.

Table S2. Interview guide used for interviews with living lab project leaders.

**English version**
**Introduction**
1. Overall, how do you feel about the implementation in the X living lab?
**Reach**

Key questions

1. Which patients were reached, and were these the patients that you had intended to reach?

Prime questions

2. Which patients (groups) turned out to be more difficult to reach?
3. Would you use different selection criteria if you were to do it again? If so, what adjustments would you make?
4. To what extent was the selection of patients automated?

Effectiveness

Key questions

1. In your opinion, was the chosen intervention worthwhile for this patient group? Why or why not?

Prime questions

1. Which subgroups benefited more from the intervention, in your view? Why?
2. Were there any unintended effects for patients or healthcare providers?
3. Were certain tasks left undone or given less attention because of the time spent on the intervention?

Adoption

Key questions

1. If the intervention was not (well) adopted by a location or a specific healthcare provider, why was that? How did you deal with this?

Prime questions

1. Who were the main implementers? Were tasks sometimes delegated?
2. What were the characteristics of locations/providers that participated well versus those that did not?
3. What were important aspects that made participation easier for pharmacies?
4. Which aspects did pharmacies perceive as barriers to participation?

Implementation

Key questions

1. To what extent was the intervention carried out as intended, in your view?
2. Which intervention components are crucial?
3. Did some of you adapt or omit intervention components?
4. What were important successful elements of the implementation process? For example: activities, materials, roles and qualities of special individuals. And what were the main bottlenecks?

Maintenance

Key questions

1. What plans exist to sustain the intervention in your own setting?
2. Are there plans to scale the intervention to another setting?

Prime questions (for both of the key questions, ask the questions below)

1. What specific plans exist?
2. What barriers are there or do you expect?
3. What support do you need from Make-It or other external parties?

Closing

1. What did you think of the guidance provided by Make-It? Wat could we have done better? Do you have tips for the second round of guidance?
2. Finally, are there any topics we haven't discussed yet that you would like to share?

Dutch version

Introductie

Hoe vind je over het algemeen dat de implementatie is gegaan in proeftuin X?

Reach

Key questions

1. Welke patiënten zijn bereikt en zijn dit de patiënten die jullie graag hadden willen bereiken?

Prime questions

2. Welke patiënten(groepen) bleken lastiger te bereiken?
3. Zouden jullie andere selectiecriteria gebruiken als je het nog een keer zou doen? Zo ja, welke aanpassingen zou je doen?

In hoeverre was de selectie van patiënten geautomatiseerd?

Effectiveness

Key questions

1. Was de gekozen interventie in jouw ogen de moeite waard voor deze patiëntengroep? Waarom wel/niet? *Prime questions*
2. Welke subgroepen hebben in jouw ogen meer baat gehad bij de interventie? Waarom?
3. Waren er misschien ook ongewenste effecten bij patiënten of bij zorgverleners?

Zijn er bepaalde taken blijven liggen of hebben taken minder aandacht gekregen door de tijd besteed aan deze interventie?

Adoption

Key questions

1. Als de interventie niet (goed) werd opgepakt door een locatie of specifieke zorgverlener, waarom was dat? Hoe ben je daarmee om gegaan?

Prime questions

2. Wie waren de belangrijkste uitvoerders? Werden de taken ook wel eens gedelegeerd?
3. Wat waren karakteristieken van wel goed en niet (goed) deelnemende locaties/zorgverleners?
4. Wat waren belangrijke aspecten die deelname (beter) mogelijk maakte?

En welke aspecten werden door apotheken als belemmeringen voor deelname gezien?

Implementation

Key questions

1. In hoeverre is de interventie in jouw ogen uitgevoerd zoals beoogd?
2. Welke interventie onderdelen zijn cruciaal ?
3. Hebben (sommigen van) jullie onderdelen aangepast of weggelaten? Waarom?

Wat zijn belangrijke succesvolle elementen van het implementatie proces? Bijvoorbeeld activiteiten, materialen, rollen en kwaliteiten van specifieke personen? En wat zijn knelpunten?

Maintenance

Key questions<br>1. Welke plannen zijn er om de interventie te borgen in jullie eigen setting?<br>2. Zijn er plannen om de interventie op te schalen naar een andere setting?<br>*Prime questions* (voor beide vragen stellen)<br>3. Wat voor concrete plannen zijn er?<br>4. Welke barrières zijn er of verwachten jullie daarbij?<br>Welke ondersteuning hebben jullie daarbij nodig vanuit Make-It? Of andere externe partijen?
**Afsluiting**
1. Wat vonden jullie van de begeleiding van Make-It? Wat hadden wij beter kunnen doen? Hebben jullie tips voor de tweede ronde begeleiding?<br>2. Zijn er tot slot nog zaken waar we het nog niet over hebben gehad, die jullie graag kwijt willen?

Note: this interview guide was designed to gather qualitative information about the implementation process in the living labs by using the RE-AIM domains, rather than solely to identify implementation determinants. Implementation determinants were also captured in the interviews as they help explain the implementation outcomes.

Table S3. Evaluated CFIR constructs not included in tailored framework.

**Innovation**
Innovation Source
Innovation Evidence Base
Innovation Relative Advantage
Innovation Trialability
Innovation Complexity
Innovation Cost
**Outer Setting**
Partnerships & Connections
Policies & Laws
Financing
Market Pressure
Performance-Measurement Pressure
**Inner Setting**
Relational Connections
Recipient Centeredness
Learning Centeredness
Tension for Change
Incentive Systems
Mission Alignment
**Individuals – Roles**
Mid-Level Leaders
Opinion Leaders
Implementation Leads (not formally appointed)
Innovation Recipients
**Individuals – Characteristics**
Need (patient)

Capability (self-efficacy)
Motivation (leaders)
